# A Pelagic Microbiome (Viruses to Protists) from a Small Cup of Seawater

**DOI:** 10.3390/v9030047

**Published:** 2017-03-17

**Authors:** Flavia Flaviani, Declan C. Schroeder, Cecilia Balestreri, Joanna L. Schroeder, Karen Moore, Konrad Paszkiewicz, Maya C. Pfaff, Edward P. Rybicki

**Affiliations:** 1 Department of Molecular and Cell Biology, University of Cape Town, Private Bag X3, Rondebosch 7701, South Africa; flafla@mba.ac.uk; 2 Marine Biological Association of the UK, Citadel Hill, Plymouth PL1 2PB, UK; cecilia.balestreri@gmail.com (C.B.); joanna.schroeder.uk@gmail.com (J.L.S.); 3 University of Exeter Sequencing Service, Biosciences, Stocker Rd., University of Exeter, Exeter EX4 4QD, UK; K.A.Moore@exeter.ac.uk (K.M.); K.H.Paszkiewicz@exeter.ac.uk (K.P.); 4 Department of Environmental Affairs, Oceans and Coasts, P.O. Box 52126, Victoria and Alfred Waterfront, Cape Town 8000, South Africa; maya.pfaff@gmail.com

**Keywords:** microbiome, viruses, prokaryote, eukaryote, NGS, diversity, phylotypes, eDNA, meDNA

## Abstract

The aquatic microbiome is composed of a multi-phylotype community of microbes, ranging from the numerically dominant viruses to the phylogenetically diverse unicellular phytoplankton. They influence key biogeochemical processes and form the base of marine food webs, becoming food for secondary consumers. Due to recent advances in next-generation sequencing, this previously overlooked component of our hydrosphere is starting to reveal its true diversity and biological complexity. We report here that 250 mL of seawater is sufficient to provide a comprehensive description of the microbial diversity in an oceanic environment. We found that there was a dominance of the order *Caudovirales* (59%), with the family *Myoviridae* being the most prevalent. The families *Phycodnaviridae* and *Mimiviridae* made up the remainder of pelagic double-stranded DNA (dsDNA) virome. Consistent with this analysis, the Cyanobacteria dominate (52%) the prokaryotic diversity. While the dinoflagellates and their endosymbionts, the superphylum Alveolata dominates (92%) the microbial eukaryotic diversity. A total of 834 prokaryotic, 346 eukaryotic and 254 unique virus phylotypes were recorded in this relatively small sample of water. We also provide evidence, through a metagenomic-barcoding comparative analysis, that viruses are the likely source of microbial environmental DNA (meDNA). This study opens the door to a more integrated approach to oceanographic sampling and data analysis.

## 1. Introduction

The paradigm of “everything is everywhere, but the environment selects” [1] suggests that all microbial taxa have the potential to be found everywhere. This largely holds true for the main marine bacteriophage taxa, with the presence of cyanophage-like sequences of the order *Caudovirales* dominating all ocean viromes, including the recently sampled Indian Ocean [2,3,4,5]. The order *Caudovirales* is comprised of three families: *Myoviridae* (contractile tails), *Siphoviridae* (non-contractile tails) and *Podoviridae* (short tails) [6]. During the Global Ocean Sampling (GOS) expedition [5], myovirus-associated sequences were ubiquitously distributed among sampling sites with the highest prevalence in tropical oligotrophic locations. Podo- and siphoviruses showed site-specific distributions, with the highest abundances recorded in temperate mesotrophic waters and hypersaline lagoons, respectively. Within the Indian Ocean, 32% of the viral fraction (VF) was attributed to known viruses, with 95% of the known viruses identified as belonging to the order *Caudovirales* (*Myoviridae*, 54.3%; *Podoviridae*, 27.6%; *Siphoviridae*, 17%) [4]. The nucleo-cytoplasmic large DNA viruses (NCLDVs) were often the next major lineage present, with the family *Phycodnaviridae* representing 83.9% of this group, followed by *Iridoviridae* at 8.5% and *Mimiviridae* at 7.3%.

Most of the virome-based studies carried out so far do not report on the diversity of the likely hosts that the viruses infect, making it unclear as to whether the viruses present in the water column are the result of active or past infections. An exception is the Tara Oceans expedition, where eukaryotic and prokaryotic diversity [7,8] was reported in conjunction with viral diversity [9,10,11]. Global surveys, which include the southwest Indian Ocean, indicate that the α-Proteobacteria dominate the prokaryotic communities in both surface waters and at the deep chlorophyll maxima. The second most represented group are either the Cyanobacteria or γ-Proteobacteria, depending on location [8]. For the eukaryotic fraction, samples collected during the Tara Ocean expedition showed that the pico- and nano-plankton was dominated by photosynthetic dinoflagellates (of the family Dinophyceae). Parasites of the superphylum Alveolata, specifically the marine alveolates (MALV)-I and MALV-II clusters, routinely infect members of the family Dinophyceae and can account for up to 88% of the eukaryote fraction in some locations. These two MALV clusters have recently been renamed Syndiniales groups I and II, respectively [12]. Specifically for the southwest Indian Ocean, the eukaryotic fraction was dominated by alveolates including the Dinophyceae and their Syndiniales parasites [7].

Studies on microbial diversity in aquatic environments rely on sample volumes ranging from tens of litres to as much as a thousand litres of water [2,13,14]. Sampling of large volumes was thought to be a necessity for early sequencing technologies, which required considerable quantities (micrograms) of DNA. Newer technologies, such as linear amplification deep sequencing with Illumina, require smaller quantities (nanograms) of DNA [15]. Additionally, various sample concentration methods have been developed in order to collect the greatest quantities of DNA possible from water samples [16]. Standard viral filtration methods involve the use of filters with a pore size of 0.2 µm to remove bacteria from the sample and collect only the virus fraction. However, this 0.2 µm size fraction results in underreporting of giant viruses [17,18], as the giant virus particles can have diameters varying from ~0.2 to 1.5 µm, with *Pithovirus sibericum* being the largest known member of this group [19]. In addition, the <0.2 µm size fraction also contains large amounts of dissolved DNA. Jiang and Paul concluded that, in this size fraction, viral particles makes up only a small component of the filterable DNA, the majority being dissolved DNA of bacterial and eukaryotic origin [20].

Dissolved DNA forms part of environmental DNA (eDNA), derived from cellular debris produced from biota living in that environment [21]. Therefore, eDNA is being used as a tool to determine whether an invasion has taken place [22] or to track an endangered species [23]. The size fraction used to describe eDNA is the size fraction that removes larger eukaryotes (passing through a 0.5 mm mesh) but retains microbes (>0.45 µm filter). Therefore, the eDNA concept excludes the microbial community as they are retained in this size range. To our knowledge no study has yet addressed the question of whether the <0.45 µm size fraction, the microbial environmental DNA (meDNA) fraction, can be used as a proxy to describe the complete biota in any given environment.

In this study, we tested the hypothesis that the volume equivalent to a cup of seawater (250 mL) is sufficient to describe the most abundant microbial taxa (from viruses to protists) in the marine environment. Serendipitously, our study site is within 548 nautical miles of station 64, previously sampled by the *Tara* Oceans expedition (−29.5333, 37.9117), thereby allowing for a semi-qualitative comparison to be made. Our protocol differed from previous studies, including that of *Tara* Oceans, as it contained no concentration steps. In addition, only 50 mL of the 0.45 µm 250 mL permeate was used to describe the combined dissolved DNA and viral fraction (meDNA). The 0.45 µm size fraction was chosen because we wanted to limit the removal of giant viruses. Here we report how a relatively small water sample can be used to capture the dominant microbial taxa within any given aquatic system.

## 2. Materials and Methods

### 2.1. Sample Collection

**Figure 1:**
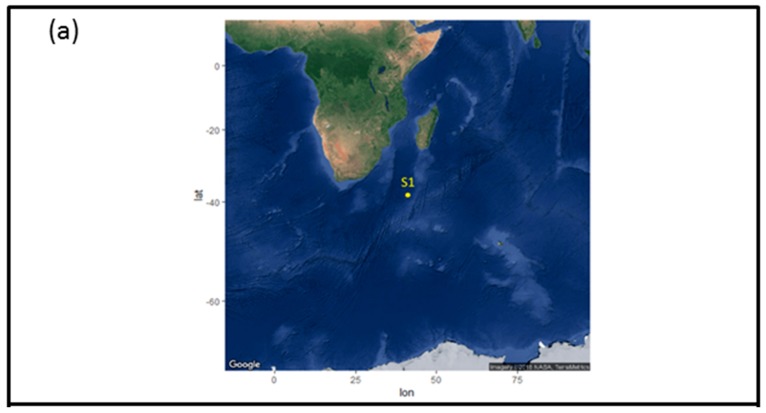

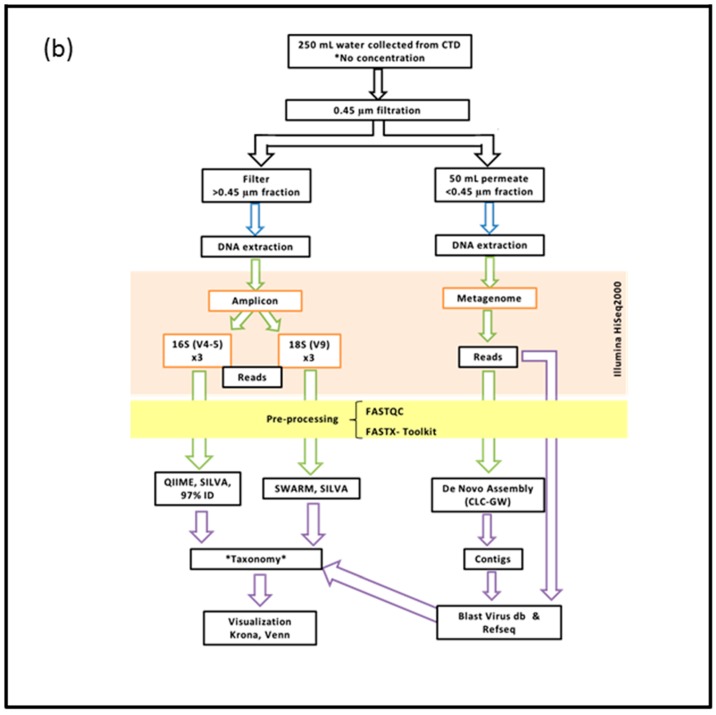
(**a**) Map showing the location of sample collection; (**b**) schematics of the bioinformatics pipeline.

The water sample analysed in this study was collected during the second transect of the Great Southern Coccolithophore Belt expedition (GSCB-cruise RR1202) in the southwest Indian Ocean in February 2012 [24]. The location of the sampling station S1 (−38.314983, 40.958083, water temperature 20.83 °C, pH 8.08) was mapped using RgoogleMaps_1.2.0.7 [25] under R version 3.3.0 (accessed on 3 May 2016) (Figure 1a).

One litre of water was gathered from the conductivity, temperature, and depth (CTD) rosette sampler from the chlorophyll maximum layer (5 m). Of this, an aliquot of 250 mL of seawater was filtered through a 0.45-µm polycarbonate filter and the filter was used for the DNA extraction onboard the R/V Roger Revelle using Qiagen DNeasy Blood and Tissue protocol (Qiagen, Valencia, CA, USA). The DNA was stored at −21 °C and subsequently transferred to Plymouth, UK, for further processing. Fifty millilitres of filtered water were set aside, wrapped in tin foil and stored in a fridge. This too was returned to Plymouth, UK, for further processing.

### 2.2. DNA Extraction, Preparation and Sequencing of the >0.45 µm Fraction

The V4 region, along the prokaryotic 16S ribosomal RNA gene was amplified using the universal primer pair 515F and Illumina tagged primer 806R7, 806R10 and 806R15 (Illumina, San Diego, CA, USA) [26]. For eukaryotic 18S ribosomal RNA gene, we used the primer pair 1391F and Illumina tagged EukB6, EukB16 and EukB23 to amplify the V9 region [27]. For all polymerase chain reaction (PCRs), we added 1–5 μL of the eDNA (concentration range from 1.47 to 32.51 ng/μL), to 5X Colourless GoTaq Flexi Buffer (Promega, Madison, WI, USA), 1.5 μL MgCl_2_ Solution 25 mM, 2.5 µL dNTPs (10 mM final concentration), 1 μL Evagreen Dye 20X (Biotium, Fremont, CA, USA), 0.1 μL GoTaq DNA Polymerase (5u/μL) and 12.9 µL of sterile water for a final volume of 25 μL for each reaction. This was done to determine the mid-exponential threshold of each reaction, ran on a Corbette Rotor-Gene™ 6000 (Qiagen). The real-time PCR proceeded with an initial denaturation at 94 °C for 3 min, followed by 40 cycles of a three-step PCR: 94 °C for 45 s and 50 °C for 60 s and 72 °C for 90 s. The fluorescence was acquired at the end of each annealing/extension step on the green channel. The cycle threshold of the amplification in the exponential phase was recorded for amplification.

A second standard PCR amplification was carried out in triplicate and run at the same conditions, excluding the addition of the Evagreen Dye. The sample was removed from the machine when it reached the cycle threshold, as previously determined. Products were run on a 1.4% agarose gel to confirm the success of the amplification and the product size of the amplification. The bands were cut out and purified using the Zymoclean Gel DNA Recovery Kit (Zymo Research, Irvine, CA, USA). Quantity and quality was verified on the NanoDrop 1000 (Thermo Scientific, Wilmington, DE, USA) and QuantiFluor E6090 (Promega). V4-16S and V9-18S were prepared mixing an equimolar concentration of each amplicon triplicate into the pool for which concentration was checked on the Bioanalyser (Agilent Technologies, Santa Clara, CA, USA). The final pooled samples were denatured and diluted to 6 pM and mixed with 1 pM PhiX control (Illumina), read 1 sequencing primer was diluted in HT1, before the flowcell was clustered on the cBOT (Illumina). Multiplexing sequencing primers and read two sequencing primers were mixed with Illumina HP8 and HP7 sequencing primers, respectively. The flowcell was sequenced (100 PE) on HiSeq 2000 using sequencing by synthesis (SBS) reagents (Version 3.0). The raw sequences are available at the European Nucleotide Archive (ENA) under accession number PRJEB16346 and PRJEB16674.

### 2.3. DNA Extraction, Preparation and Sequencing of the <0.45 µm Fraction

The whole 50 mL permeate was used in the nucleic acid extraction procedure. We added 100 μL of proteinase K (10 mg/mL; Sigma-Aldrich, St. Louis, MO, USA) and 200 μL of 10% sodium dodecyl sulfate (SDS) (Sigma-Aldrich) to the permeate and incubated the solution for two hours with constant rotation at 55 °C. The lysate was then collected through multiple centrifugations on a Qiagen DNeasy Blood and Tissue column (Qiagen). The standard Qiagen protocol was followed with 20 µL nuclease-free water (Sigma-Aldrich) used as the elution agent. Quantity and quality was determined using the NanoDrop 1000 (Thermo Scientific) and QuantiFluor E6090 (Promega). Two hundred microliters of DNA (<40 ng) were fragmented using a Bioruptor (Diagenode, Seraing (Ougrée), Belgium) on medium for 15 bursts of 30 s with a 30 s pause and concentrated to 30 µL on a Minelute column (Qiagen). Fragments were made into libraries using the Nextflex ChipSeq library preparation kit (BIOO scientific, Austin, TX, USA) without size selection and with 18 cycles of PCR amplification. Bioanalyser (Agilent Technologies) analysis indicated the final library contained insert between 30 basepairs (bp) to 870 bp. The library was multiplexed with other samples and sequenced (100 paired end) on a HiSeq 2000 (Illumina) using RTA1.9 and CASAVA1.8.

### 2.4. Bioinformatics Pipeline for the Prokaryotic (16S) and Eukaryotic (18S) Amplicon

The complete bioinformatics pipeline is illustrated in Figure 1b. The read quality was first assessed using Fast-QC [28]. FASTX-Toolkit [29] was utilised for the trimming and filtering steps; the first and last 10 bases were trimmed in order to remove low quality nucleotides. Reads were then filtered in order to retain only reads with more than 95% of nucleotide positions called with a quality score of 20. Trimmed and cleaned reads from each of the triplicate V4-16S and V9-18S PCRs were pooled in order to assign OTUs using Qiime [30] with 97% similarities for clustering and Swarm analysis [31], respectively. A taxonomy was assigned using BLASTn implemented in Qiime and Swarm using SILVA Version 119 [32] with a minimum e-value of 1 × 10^−5^.

### 2.5. Bioinformatics Pipeline of the <0.45 µm Fraction (Metagenome)

As for the amplicon dataset, the quality of the reads was first assessed using Fast-QC [28]. The FASTX-Toolkit [29] was used to trim the first last bases to remove low quality nucleotides, and subsequently to filter out reads with fewer than 95% of nucleotide positions called with a quality score of 20. The forward read (R1) of the 100 bp pair-end HiSeq reads have been subjected to random library size normalization using Qiime script subsample_fasta.py; reverse reads (R2) had poor quality and were therefore discarded. The reads were used in a BLASTX [33] analysis against a Virus database (db; courtesy of Pascal Hingamp) with e-values less than 1 × 10^−5^. The Virus database consisted of Refseq curated viral genomes, together with additional new genomes [11], and 20% of R1 Refseq whole organism db [34]. In addition, the pair-end reads were assembled into contigs using a de Bruijn de novo assembly program in CLC Genomic Workbench (Version 7.1.5; CLCbio, Cambridge, MA, USA) using global alignment with automatics bubble and word size, minimum contigs length of 250, mismatch cost of 2, insertion and deletion cost of 3, length fraction of 0.5 and similarity threshold of 0.8. The contigs were annotated with the BLASTX as described for the R1 normalised reads. Blast analyses were performed by using the University of Cape Town’s HPC hex cluster.

The top hits from all the blast searches were selected through the use of a parser Perl script (http://www.bioinformatics-made-simple.com), and then a customised R script was developed to assign taxonomy. A complete viral taxonomy was assigned through a manually curated implementation of the International Committee on Taxonomy of Viruses (ICTV) database 2013 v1 with the National Center for Biotechnology Information (NCBI) taxonomy database.

### 2.6. Visualization of Community Diversity

Krona tools [35] were used to visualize community diversity as characterized by the Silva (v119), Refseq and Virus db genes taxonomy assignments. Venn diagrams were created using the R package VennDiagram_1.6.17 on R (Version 3.3.0; 2016-05-03).

### 2.7. Filters Applied to Annotated Datasets

We performed independent analyses on three independent PCR replicates (V4-16S and V9-18S) and assigned a taxonomy using Silva [36]. By using replication, we removed the level of noise in the sample introduced by PCR and sequencing artefacts, while retaining rare organisms. Therefore, we considered four levels of stringency at the phylotype level: (1) T0, all phylotypes present across the three replicates; (2) T1, removing singletons from each replicate; (3) T10, a minimum of 10 copies per phylotype had to be present in any one of the replicates, (4) T10-R1, a minimum of 10 copies per phylotype present in any two replicates and (5) T10-R2, a minimum of 10 copies per phylotype present in all three replicates.

## 3. Results

### 3.1. Microbiota in the >0.45 µm Fraction

**Figure 2:**
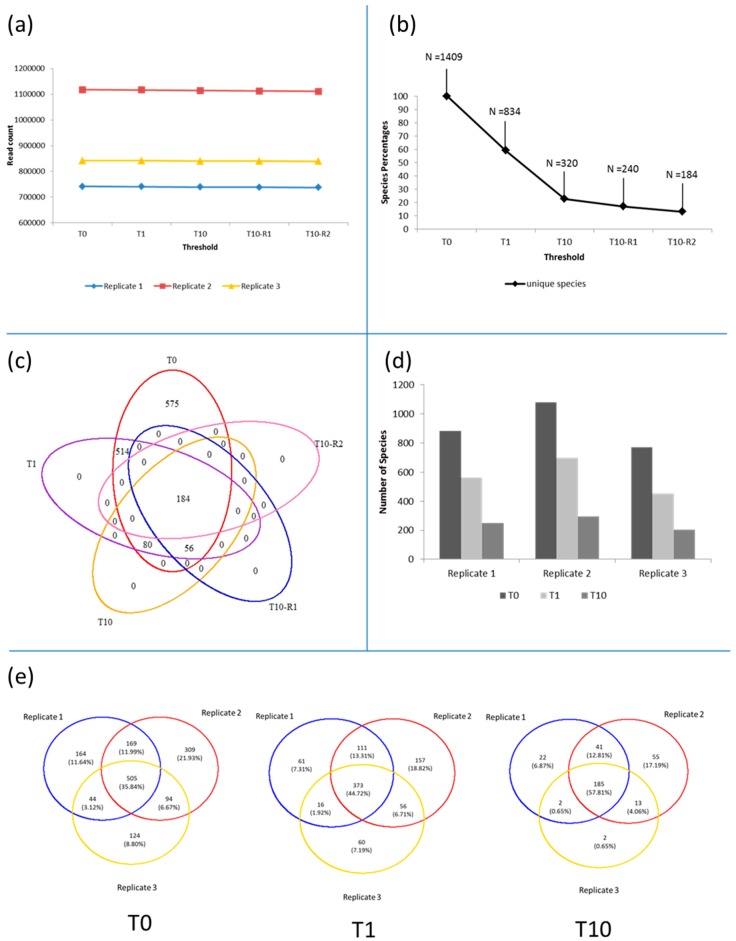
Analyses of the prokaryotic fraction. (**a**) Reduction in number of reads when filters are applied; (**b**) percentage and phylotype count when filter are applied; (**c**) presence–absence analyses at phylotype level before and after application of the filters; (**d**) number of phylotype analyses by replicate; (**e**) presence–absence analyses at phylotype level when filters are applied to each replicate.

**Figure 3:**
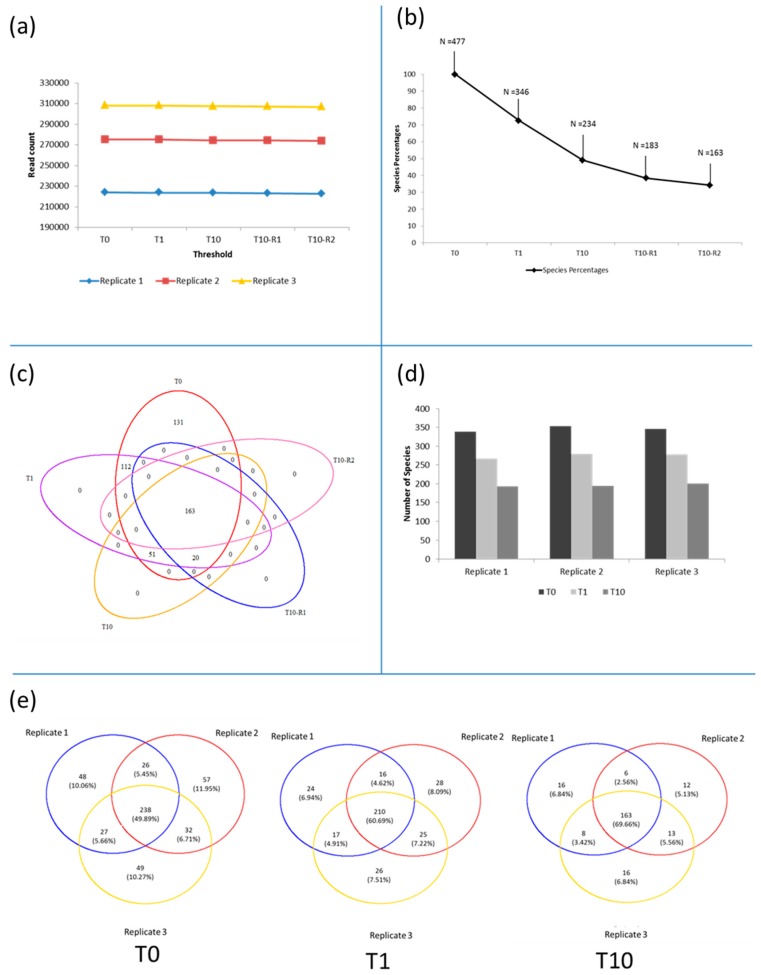
Analyses of the eukaryotic fraction. (**a**) Reduction in number of reads when filters are applied; (**b**) percentage and phylotype count when filters are applied; (**c**) presence–absence analyses at phylotype level before and after application of the filter; (**d**) number of phylotypes analyses by replicate; (**e**) presence–absence analyses at phylotype level when filters are applied to each replicate.

After pre-processing, which included a specific subsampling to an equal read length of 125 bases, we retained an average 0.9 million reads for the prokaryotic and around 270 thousand for the eukaryotic dataset (Table 1). These reads clustered (T0 applied to combination of the three replicates) into around 46 thousand unique Operational Taxonomic Units (OTUs) for the prokaryotes, which clustered into 1409 phylotypes. For the eukaryotes 6836 OTUs clustered into 477 phylotypes (Table 1). Four different filters were applied which resulted in an increase in selection stringency (T0 to T10-R2) without the removal of significant numbers of reads from the prokaryotes (Figure 2a) and eukaryotes (Figure 3a) datasets, independent of sequence depth. However, the greatest change observed due to the application of the filters, was seen in the number of phylotypes observed (Figure 2b and Figure 3b). A total number of 1886 phylotypes was observed in the 250 mL of southwest Indian Ocean, made up of 1409 prokaryotic and 477 eukaryotic phylotypes. When the singletons were removed (T1), the number of prokaryotic phylotypes dropped by nearly a half to 834 (59.19%, phylotypes retained) (Figure 2b); this was also observable in the OTUs (Table 1) moving from 45,826 to 23,081. Similarly, the number of eukaryotic phylotypes dropped by a third to 346 phylotypes (72.54% phylotypes retained) (Figure 3b), whilst OTUs dropped from 6836 to 2930 (Table 1). When a further filter, that a minimum of at least 10 reads per phylotype must be present in any of the replicates (T10), was applied, the diversity dropped by an additional 36% (compared to T0) to just under 77% for prokaryotes—retaining only 23% (Figure 2b), and 24% to 51% in eukaryotes—retaining only 49% (Figure 3b), leaving a total number of phylotypes as 554.

The phylotypes removed after applying the singleton filter (T1) (Supplementary Table S1) included *Cicer arietinum* (chickpeas), *Sesamum indicum* (sesame) and *Nicotiana sylvestris* (tobacco), which were not expected to be present in the marine environment. The application of the T10 filter resulted in the removal of a few marine species instead, such as *Noctiluca scintillans*, *Amphidinium mootonorum* and *Pandorina morum*. The additional application of replication filters, present in greater than 10 copies in at least any two (T10-R1) and all three (T10-R2) replicates, revealed a further but minimal reduction in the overall phylotype content (Figure 2b and Figure 3b): both the prokaryotes and eukaryotes dropped to 17% and 38% (from T10 to T10-R1, Figure 2b) and 13% and 34% (from T10 to T10-R2, Figure 3b), respectively. We could identify a core of 184 phylotypes for the prokaryotes (Figure 2c) and 163 for the eukaryotes (Figure 3c) which were common across all filters. If no filter was applied, 575 prokaryotes (41%) and 131 eukaryotes (27%) unique or rare were observed, however, irrespective of which filter is applied no phylotype unique to their stringency were observed (Figure 2c and Figure 3c).

In summary, we have identified a total of 1886 phylotypes of prokaryotes and eukaryotes without the application of any filter (T0), which was reduced to 1,180 after singletons were removed (T1). A further decrease in phylotype composition to 554, 423 and 347 was identified after application of T10, T10-R1 and T10-R2 filters.

We then considered the three replicates independently in order to understand how phylotypes differ across the three PCR replicates (Figure 2d and Figure 3d). Prokaryotic diversity ranged from 767 phylotypes in replicate 3 to 1077 in replicate 2 (Figure 2d), corresponding to the sequence depth (Figure 2a). This was however not observed for the eukaryotes (Figure 3d), ranging from 339 of replicate 1 to 353 of replicate 2 (Figure 3d), irrespective of the sequence depth (Figure 3a). When applying the T1 filter, the number of phylotypes retained were on average 65% (from 882 to 561 in replicate 1, from 1077 to 697 replicate 2 and from 767 to 505 in replicate 3) and 79% (from 339 to 267 in replicate 1, from 353 to 279 in replicate 2 and from 346 to 278 in replicate 3) of the prokaryotes and eukaryotes, respectively (Figure 2d and Figure 3d). Applying stringency filter T10 reduced the prokaryotic diversity in replicate 1 to 28%, in replicate 2 to 27% and replicate 3 to 26% (Figure 2d), whilst for the eukaryotes across replicates 1, 2 and 3 to 57%, 55% and 58%, respectively (Figure 3d).

Phylotype composition at T0 had 36% prokaryotic and 50% eukaryotic phylotypes in common across all replicates (Figure 2e and Figure 3e). Between 9% and 22% of prokaryotes and 10% and 22% of eukaryotes were unique to each replicate. When singletons (T1) were removed and the T10 filter applied, the phylotypes common across all replicates increased to 45% and 58% for prokaryotes (Figure 2e), whilst for the eukaryotes, increased to 61% and 70% (Figure 3e). This coincided with the reduction in unique phylotypes retained per replicate. Replicate 1, 2 and 3 changing from 164 to 22, 309 to 55 and 124 to 2 unique prokaryotic phylotypes (Figure 2e). Similarly, replicate 1, 2 and 3 changed from 48 to 16, 57 to 12 and 49 to 16 unique eukaryotic phylotypes (Figure 3e).

### 3.2. Diversity and Community Structure of the >0.45 µm Fraction

Cyanobacteria made up 42% of the prokaryotic community diversity; their composition was dominated by the genera *Synechococcus* (30%) and *Prochlorococcus* (9%) (Figure S1). The V4-16S universal primers also amplified the eukaryote plastid ribosomal genes, making up 2.68% of the total sequences. The second most diverse bacterial group were the Proteobacteria (32%), comprising the orders α-Proteobacteria (20%), γ-Proteobacteria (8%) and δ-proteobacteria (3%). The α-Proteobacteria comprised the orders Rhodospirallales (5%), SAR11 clade (5%), Rickettsiales (5%), Rhodobacteriales (4%) and the OCS116 clade (0.4%). The γ-Proteobacteria comprised the orders Oceanospirallales (6%), Alteromonadales (0.8%), Marinicella (0.7%) and K189A clade (0.5%). The δ-Proteobacteria were assigned to the SAR324 clade (3%). Bacteroidetes and Actinobacteria represented 4% and 2% of the prokaryote diversity. Finally, a large component (20%) of the prokaryotic community could not be assigned to any known sequences (Figure S1).

The eukaryotic community was dominated (92%) by the superphylum Alveolata (Figure S2), comprising the Protoalveolata (44%), Dinoflagellata (40%), Ciliophora (3%) and FV18-2D11 (3%). Protoalveolata were dominated by Syndiniales (97%), subdivided as: Group II (57%), Group I (18%), Amoebophyra (17%), Duboscquella (4%) and Perkinsidae (3%). The group Dinoflagellata was formed by Peridiniphycidae (16%), Gymnodiniphycidae (14%), Dinophysiales (1%) and Prorocentrum (0.7%).

### 3.3. Diversity of the <0.45 µm Fraction

**Figure 4:**
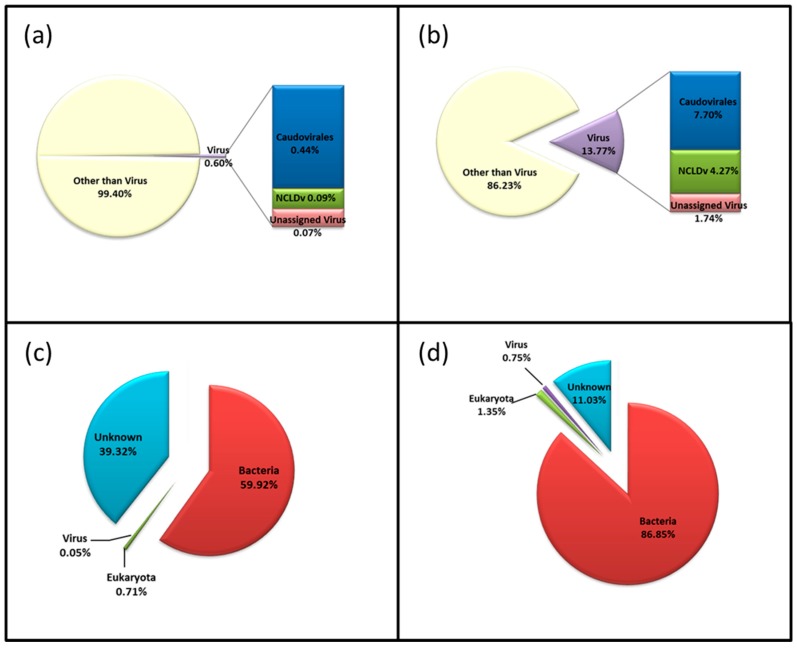
Taxonomic assignment based on reads (**a**,**c**) and contigs (**b**,**d**) analyses. Reads (R1) were annotated using (**a**) the Virus database and (**c**) the Refseq database; contigs were annotated using (**b**) the virus database and (**c**) the Refseq nr-protein database.

After pre-processing 10 million paired reads were assembled to contigs with an average contig length of 1045 bp (Table 1), and a subsample of 1.5 million reads from R1 were utilised for analyses at the level of reads. The majority of sequences and predicted genes based on BLASTX against a virus database could be annotated as “other than virus” (Figure 4a). This was independent of whether the reads (99%) or the assembled contigs (86%) were used for the annotation (Figure 4b). Using the Refseq database, the metagenome could be divided into 59.92% Bacteria, 39.32% unknown, 0.71% Eukaryota and 0.05% Viruses at the read level, whilst for the contigs the hits could be divided into Bacteria (86.85%), unknown (11.03%), Eukaryota (1.35%), Viruses (0.75%) and Archaea (0.02%) (Figure 4c,d).

**Figure 5:**
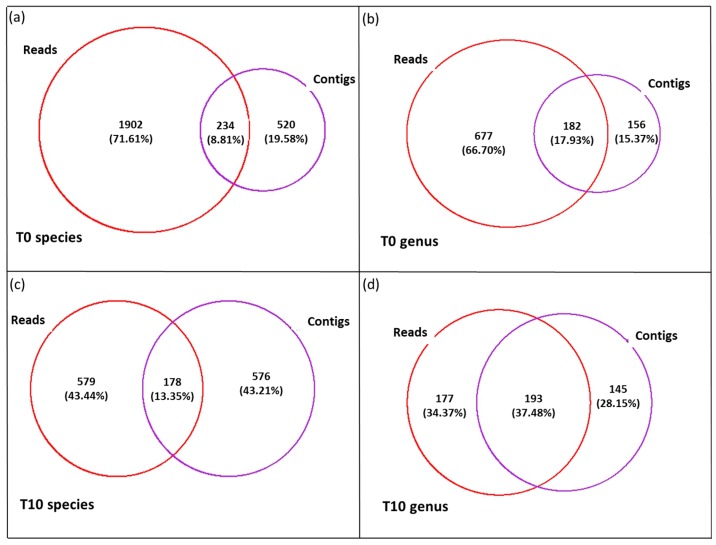
Presence–absence analyses of the <0.45 µm fraction. Comparison of phylotypes at the level of species (**a**,**c**) and genus (**b**,**d**) using a subsample of reads (R1) versus contigs at T0 (**a**,**b**) and T10 (**c**,**d**).

Utilizing the output from the Refseq database we compared annotation based on reads versus contigs. We observed very low similarities between the phylotypes annotated in the reads compared to the contigs (Figure 5). Only 8.81% of phylotypes were common across the two methods when no filter was applied (T0; Figure 5a), whereas 13.35% were common when T10 was applied (Figure 5c). To account for the high level of randomness associated with the top hits from BLAST outputs especially from universal conserved genes, we repeated the analyses using a lower stringency annotation, i.e., the genus as lowest level of classification instead of the phylotypes. Common annotations between the analysis based on reads versus contigs increased to 17.93% at T0 (Figure 5b) and 37.48% at T10 (Figure 5d). Therefore, from here on we focused our attention on the annotation based on the contigs. The Refseq annotation (Figure S3) produced an output highly dominated by Actinobacteria (47%) and Proteobacteria (38%). Specifically, the order Microcroccales made up 41% of sequences with the genus *Microbacterium* being the most dominant (33% of all the bacteria). The Proteobacteria comprised the classes’ α-Proteobacteria (37%), γ-Proteobacteria (1%) and β-Proteobacteria (0.5%). The class α-Proteobacteria was dominated by the order Sphingomonadales (33%), with the genus *Erythrobacter* representing 24% of all the contigs, for which one coding sequence matched a 16S gene (Figure S3). Eukaryotes were represented in 1.35% of the metagenomic fraction and were dominated by the family Phaeophyceae (87%). Metazoa constituted only 0.07% of the eukaryotes (Figure S4).

**Figure 6:**
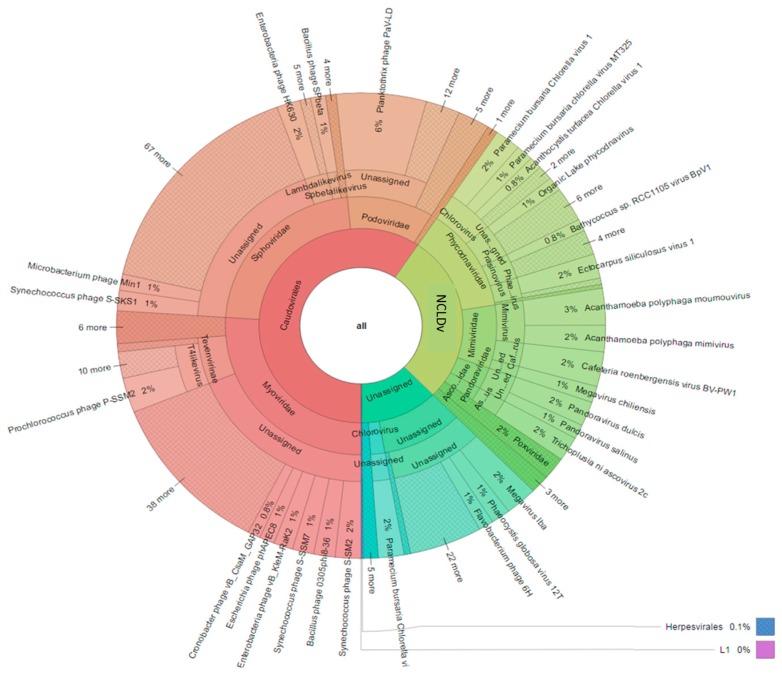
Krona chart of contigs annotation using the Virus db.

The viral contigs were further annotated using a curated Virus database (Figure 6). The virome was dominated by the order *Caudovirales* (59%) comprising the families *Myoviridae* (26%), *Siphoviridae* (22%) and *Podoviridae* (10%). The NCLDVs (28%) represented the second major order, with the families *Phycodnaviridae* (13%) and *Mimiviridae* (8%) as the main representatives.

### 3.4. Composition of Biota of the <0.45 µm versus the >0.45 µm Fraction

**Figure 7:**
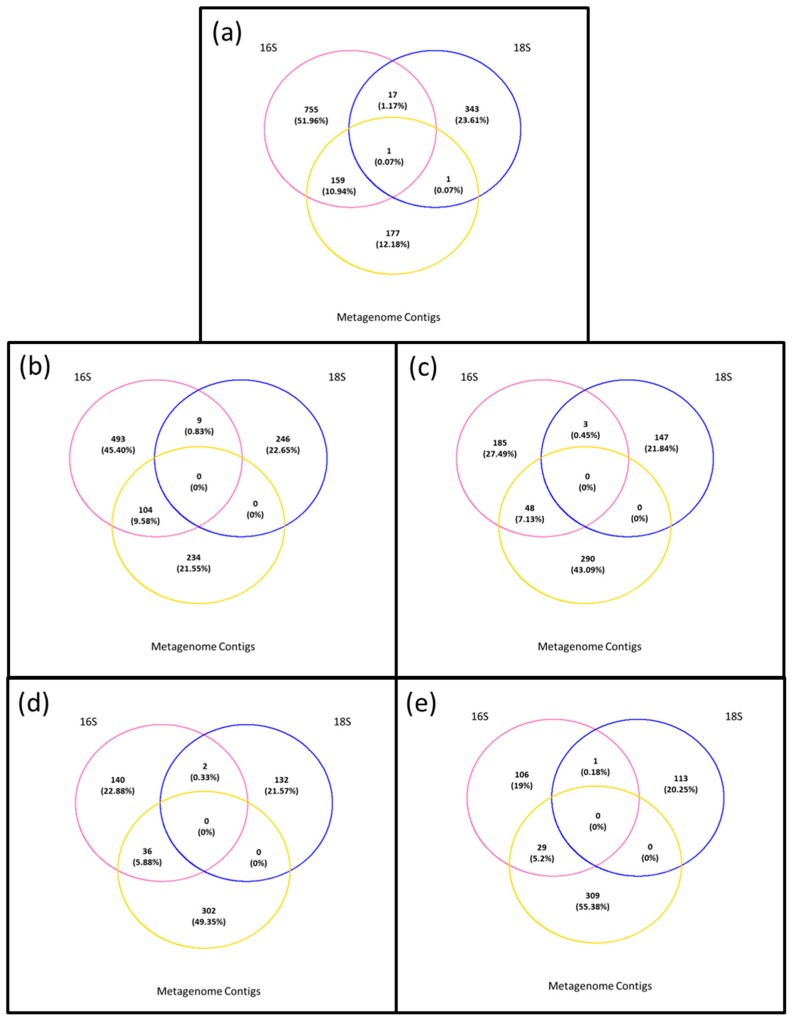
Presence–absence analyses between the >0.45 µm fraction (prokaryotes and eukaryotes) and the permeate (<0.45 µm). (**a**) T0: Metagenomic contigs, prokaryotes, eukaryotes; (**b**) T0: Metagenomic contigs, T1: prokaryotes, eukaryotes; (**c**) T0: Metagenomic contigs, T10: prokaryotes, eukaryotes; (**d**) T0: Metagenomic contigs, T10-R1: prokaryotes, eukaryotes; (**e**) T0: Metagenomic contigs, T10-R2: prokaryotes, eukaryotes.

To understand if the prokaryotes and eukaryotes identified in the permeate (<0.45 µm) consisted of environmental DNA (debris or vesicles from extant Bacteria and Eukaryotes present in the water column), stable free DNA, or small Bacteria that passed through the filter, we ran presence–absence analyses comparing presence of microbiota in the <0.45 µm versus the >0.45 µm fraction for each filter (Figure 7). We also ran the analysis at the genus level or, when the genus annotation was not available, at the highest taxonomic level available. Very little overlap was observed across all levels of stringency (Figure 7). The genus *Phaeodactilum* (Table S1), shared between all datasets at T0, disappeared when singletons were removed (Figure 7b). Commonalities between eukaryotes and prokaryotes showed the presence of chloroplasts and mitochondria in the prokaryotic fraction with genera shared for 1.24% at T0, 0.83% at T1 and 0.45% at T10 (Figure 7). When the filter T1 was applied, it caused the removal of unusual genera such as *Cicer*, *Cucumis*, and *Porphyridium*, whilst genera such as *Chlorella*, *Chroomonas*, *Karlodium* and *Pedinomonas* disappeared with T10 filter (Table S2).

## 4. Discussion

Microbes, from the smallest viruses to the largest unicellular protists, dominate our oceans, playing a central role in ocean food webs and as key drivers of biogeochemical processes [37]; yet the complex interactions and ecological significance of these relationships within and between biomes are largely unknown. The necessity of studying prokaryotes, eukaryotes and viruses together was highlighted in 2011 when it was estimated that only 11.2% and 2.2% of selected literature utilised two or three microbial groups, respectively [38]. For this reason, the more recent ocean expeditions sampling efforts include multiple trophic levels and ecosystem components in an attempt to better describe the complex microbial ecosystem structure and dynamics [39]. Describing and studying the hosts (prokaryotes and eukaryote assemblages) alongside their viruses can help improve our understanding of the roles of microbes in a more holistic way.

Given the patchiness of marine environments, changing rapidly both in time and space, the definition of a unique standard sample volume remains elusive [38]. Yet fingerprint profiles in the marine environment have shown the absence of significant difference in richness when utilizing from 10 to 1000 mL of seawater [40] as well as the low variability of the community structure when utilising more than 50 mL [41]. With this study, we used 250 mL of water, sampling the same seawater mass for all three microbial components (prokaryotes, eukaryotes and viruses). Here we demonstrate that the application of four levels of stringency allowed us to step-wise eliminate OTUs produced by sequencing errors and/or contamination. The removal of singletons resulted in the reduction of the overall phylotypes by around 700, while retaining over 99% of the reads. This step removed sequences of terrestrial origin (e.g., *Nicotiana* and *Cicer*), which are not expected to occupy the marine microbiome. Although singleton removal is a common practice, researchers do often retain these taxa under the label of “rare” microbiome. When singletons are removed in conjunction with replication of PCR runs a more stringent and precise description of the microbiota present in the environment can be obtained. This filtering step (T1 on the three replicates combined) allowed us to identify around 23,000 OTUs for the prokaryotic dataset and 3000 for the eukaryotic dataset grouping 834 and 346 as the lowest level of assigned taxa, respectively. Furthermore, the use of replication reduced the overall retained phylotypes when compared to individual replicates, because the duplicate values across the three replicates were removed, leaving only unique annotations, which constituted the dominate phylotypes of the sample. The further application of a more stringent filter, i.e., a phylotype was present with at least 10 reads in each PCR replicate, gave us the confidence that the rare microbiota were not included accidentally in the final dataset. However, this will invariably mean that genuine rare microbiota could be removed. This was the case of taxa such as *Chlorella*, *Pedinomonas*, *Marinobacter* and *Oceanicaulis*, which were removed by applying this filter level.

Bacterial composition at the location analysed by *Tara* Oceans expedition (station 64), based 548 nautical miles from ours, showed high abundance of α-Proteobacteria followed by Cyanobacteria (chloroplasts), γ-Proteobacteria and Bacteroidetes [8]. The microbial composition in our sample revealed the dominance of Cyanobacteria (*Synechococcus* and *Prochlorococcus*) followed by α-Proteobacteria, γ-Proteobacteria and Bacteroidetes. This Cyanobacteria dominance is more consistent with other viral abundance data (discussed further later on). Eukaryotes collected from *Tara* Oceans station 64 were dominated by the pico-nanoplankton, the Alveolata (Dinophyceae and Syndiniales clade MALV-I-II), followed in abundance by “other protists” [7]; our station was also dominated by Alveolata (Dinophyceae and Syndiniales). We hypothesise that the variation in composition from our station S1 and *Tara* Oceans’ station 64 can be attributed to sampling different water masses as well as different sampling seasons: *Tara* Oceans’ one was sampled in winter (July 2010), while our station S1 was collected in summer (February 2012). Given these differences, it is nonetheless remarkable how similar the microbial communities were, especially when considering the application of vastly different sampling protocols.

Analyses of the metagenomic fraction, 0.45 µm permeate, showed that annotations based on the assembled contigs lead to a more robust description of diversity. We found that the majority (86%) of our data did not match any viral genomes in our curated virus database. This was similar to what was reported by previous studies, i.e., 55% [42], 91.4% average [2], 88% [4] and 64.48% [43]. Marine viral metagenomics or metabarcoding studies currently apply various biomass or volume concentration methods before the extraction of DNA for sequencing. Such studies applied to our area of interest reported on the dominance of the order *Caudovirales*. Members of the family *Phycodnaviridae* were the second most abundant viral group, often followed by the family *Mimiviridae*. Our study demonstrated that a similar description of viral diversity is achievable from only 250 mL of seawater. The high abundance of Prochlorococcus and Synechococcus phages was consistent with the observed dominance of their host cyanobacteria genera. Both *Prochlorococcus* and *Synechococcus* co-occurred and dominated the prokaryotic dataset with 30% and 9% of the sequences. It is curious to note that the *Tara* Oceans expedition [8] did not find any barcode sequences matching extant Cyanobacteria lineages despite the high abundance of both Prochlorococcus and Synechococcus phages in this locality. The reason for this anomaly might lie in the differing methodologies applied or indeed the difference in timing of sampling. Future side-by-side methodological comparative studies might resolve the reason behind these inconsistencies.

NCDLVs, such as *Phycodnaviridae* and *Mimiviridae*, surprisingly coincided with the presence of diatoms and dinoflagellates. These taxa, which constituted more than 90% of the eukaryotic dataset, are considered the most widespread protists on earth and are known to be routinely infected by RNA viruses [44]. Nevertheless, dinoflagellates are also infected by NCLDVs [44,45] and therefore our study suggests that further undescribed host-virus relationships can occur between dinoflagellates, diatoms and NCLDVs.

The 0.45 µm permeate or meDNA contains dissolved genetic material associated with cellular derived exudates (as part of the eDNA fraction [21]), viruses [20] or indeed small bacteria [46]. The comparative analyses of the two sampled size fractions revealed that bacteria and eukaryotes identified in this environment were not the source of the entire meDNA in our sample. On average 10% and 0% of the phylotypes was found in common between the meDNA and the bacterial and eukaryote permeate fractions, respectively. The likely explanation for the source of this DNA could be either the presence of viruses carrying host genes, since host genes have been identified in viral isolates or the presence of small bacteria (>0.45 µm). The latter included genera, identified in both datasets, such as *Pseudomonas*, *Flavobacterium*, *Serratia* and *Vibrio*, which are known to pass through 0.45 µm filters [46]. Nine coding sequences of the <0.45 µm fraction had hits with 16S proteins, six of which corresponded to *Microbacterium* (data not shown), and represented the main genera identified in this fraction. Furthermore it has been shown that, in adverse conditions, *Microbacterium* can present size reduction, which allowed it to pass through 0.45 µm filters [47,48]. Viruses often acquire host genes through horizontal gene transfer and since a large proportion of genetic material with unknown identity was also described, we hypothesise that viruses are the likely source of this meDNA. Whether this will be the case for all locations and situations remain to be determined. Our hypothesis contradicts Jiang and Paul [20], possibly because of their study locations being more productive. However, their study stopped short of confirming the species identified to actually being present in their water sample. Ultimately, eDNA, or, in our case, meDNA, do not appear to be a good proxy for describing the microbes present in this body of water.

## 5. Conclusions

To our knowledge, we report for the first time that 250 mL of seawater is sufficient to provide a comprehensive description of microbial diversity made up of 834 prokaryotic, 346 eukaryotic and 254 unique virus phylotypes. Moreover, given the paucity of fully curated marine viral genomes in searchable viral databases, we hypothesise that the meDNA fraction will be of viral origin. This in turn reinforces the potential of viruses to move host DNA around and even actively increase the repertoire of functional genes within any given individual, population, community or ecosystem. Finally, we do not recommend the use of meDNA as a proxy to describe the microbiome; however, this study needs to be replicated in other scenarios and locations.

## Figures and Tables

**Table 1:** Description of sequences generated in this study.

Fraction	Reads/Contigs	Sequence Length	OTUs and Phylotypes after Applying Filter							
			T0				T1			
			OTUs		Phylotypes		OTU		Phylotypes	
**Prokaryote**	741,033	125	20,381	45,826 ^1^	882	1409 ^1^	11,341	23,081 ^1^	561	834 ^1^
	1,117,576	125	30,642		1077		16,593		697	
	841,639	125	24,756		767		13,416		505	
**Eukaryote**	223,814	125	2972	6836 ^1^	339	477 ^1^	1714	2930 ^1^	267	346 ^1^
	275,201	125	3271		353		1780		279	
	308,208	125	3470		346		1836		278	
**Metagenome**	4,962	Average 78.9 min: 240 max: 74,442 $\bar{x}$: 1045			254 virus					

^1^ sum from the three replicates with duplicates removed.

## Supplementary Materials

The following are available online at www.mdpi.com/1999-4915/9/3/47/s1. Figure S1: HTML link to the Prokaryotic Krona chart, Figure S2: HTML link to the Eukaryotic Krona chart, Table S1: 16S and 18S species lists in T0, Table S2: Common genera.
