# Supplementary figures and images for: A Pelagic Microbiome (Viruses to Protists) from a Small Cup of Seawater
Source: Viruses. 2017 Mar 17;9(3):47. doi: 10.3390/v9030047 (PMC5371802; doi:10.3390/v9030047)

[
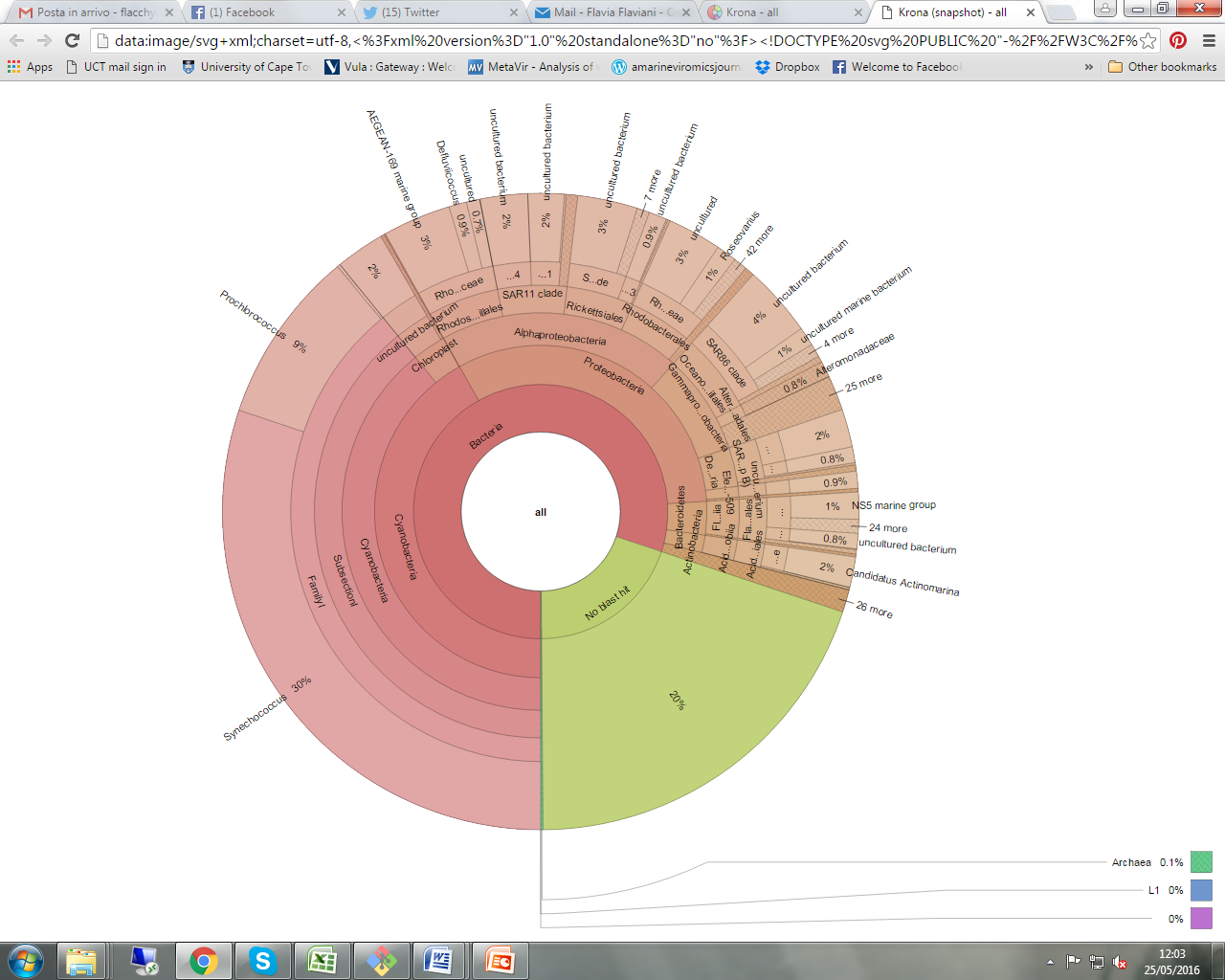
](Supplementary_figure_HTML/Supplementary_Figure1.html)

Supplement: Supplementary file 1 [file viruses-09-00047-s001.zip › Supplementary revised/Supplementary_Figure1.docx]

[
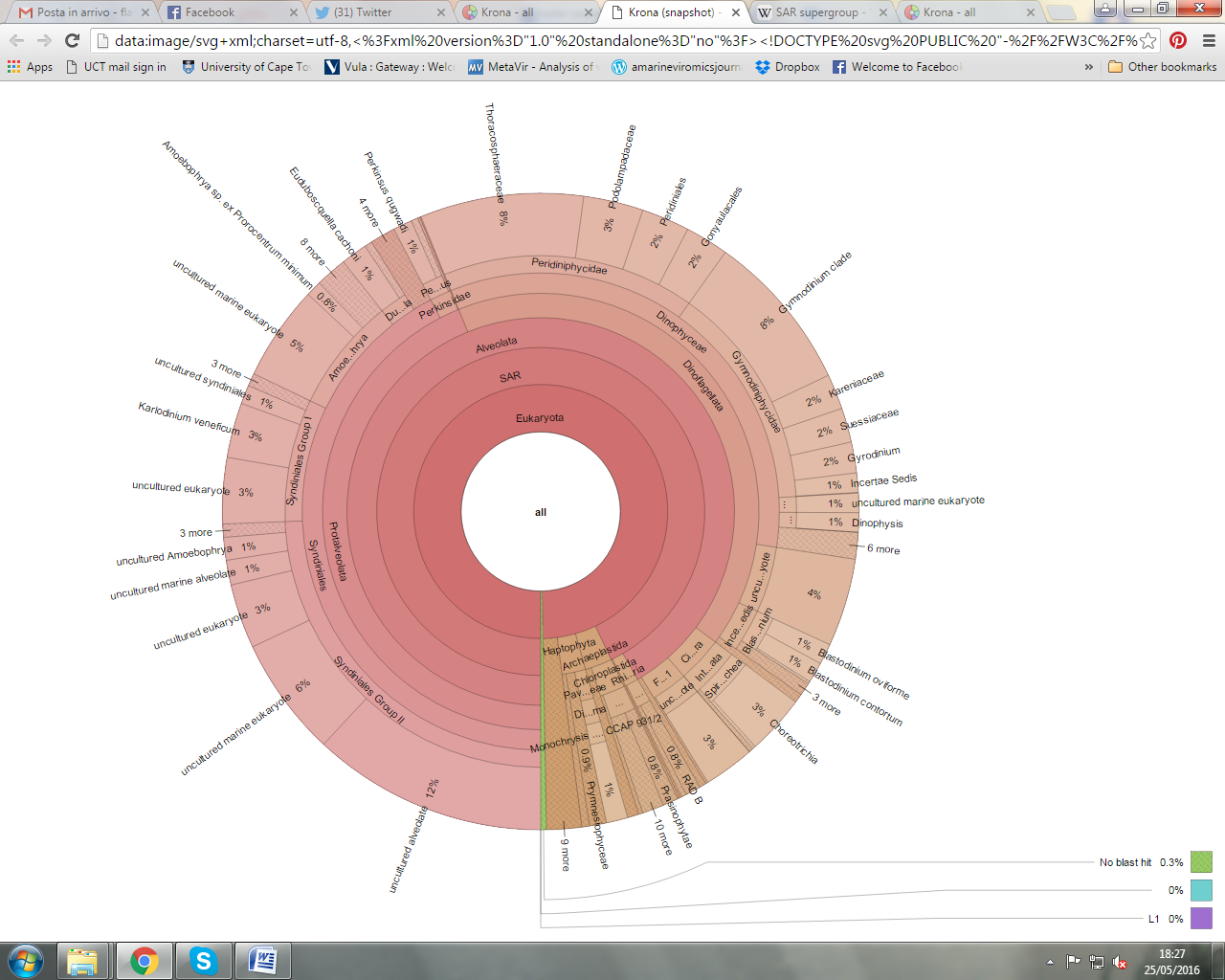
](Supplementary_figure_HTML/Supplementary_Figure2.html)

Supplement: Supplementary file 1 [file viruses-09-00047-s001.zip › Supplementary revised/Supplementary_Figure2.docx]

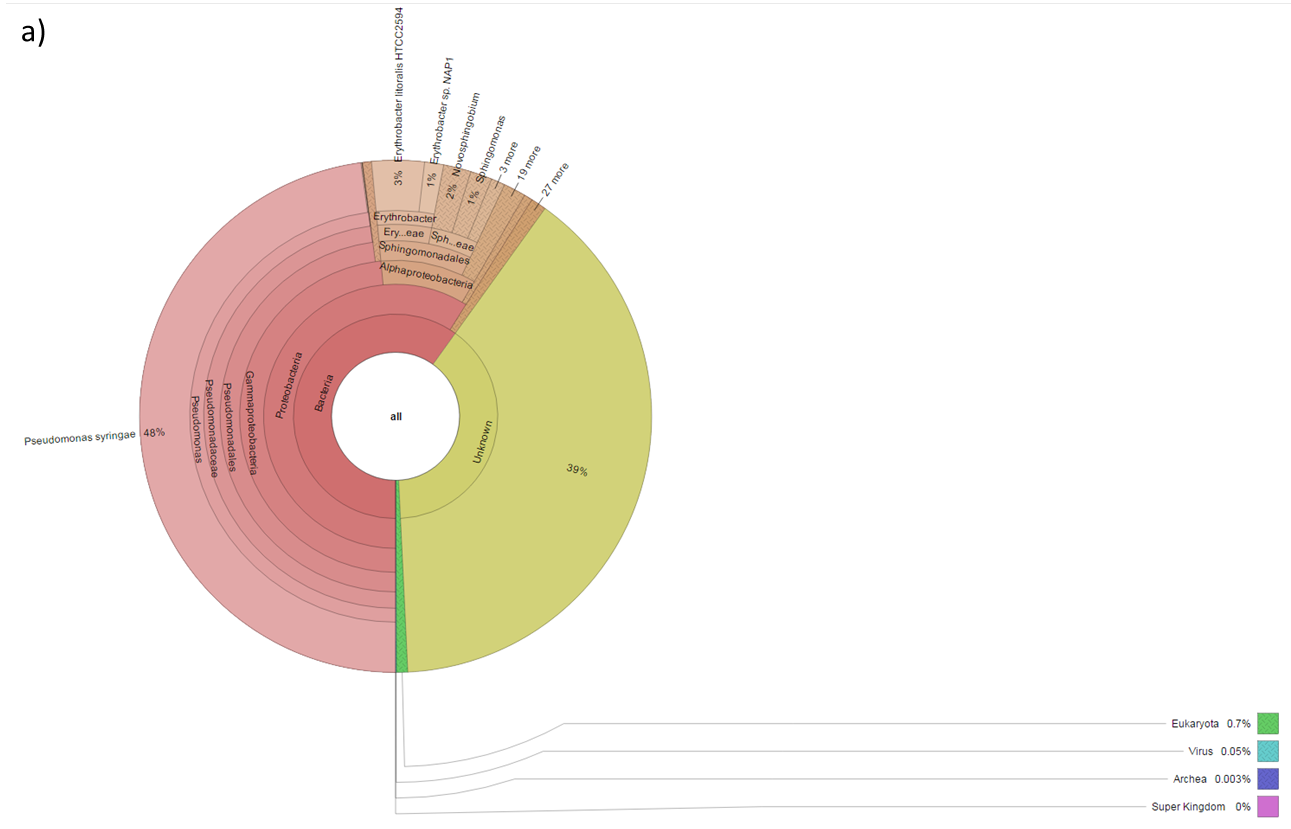


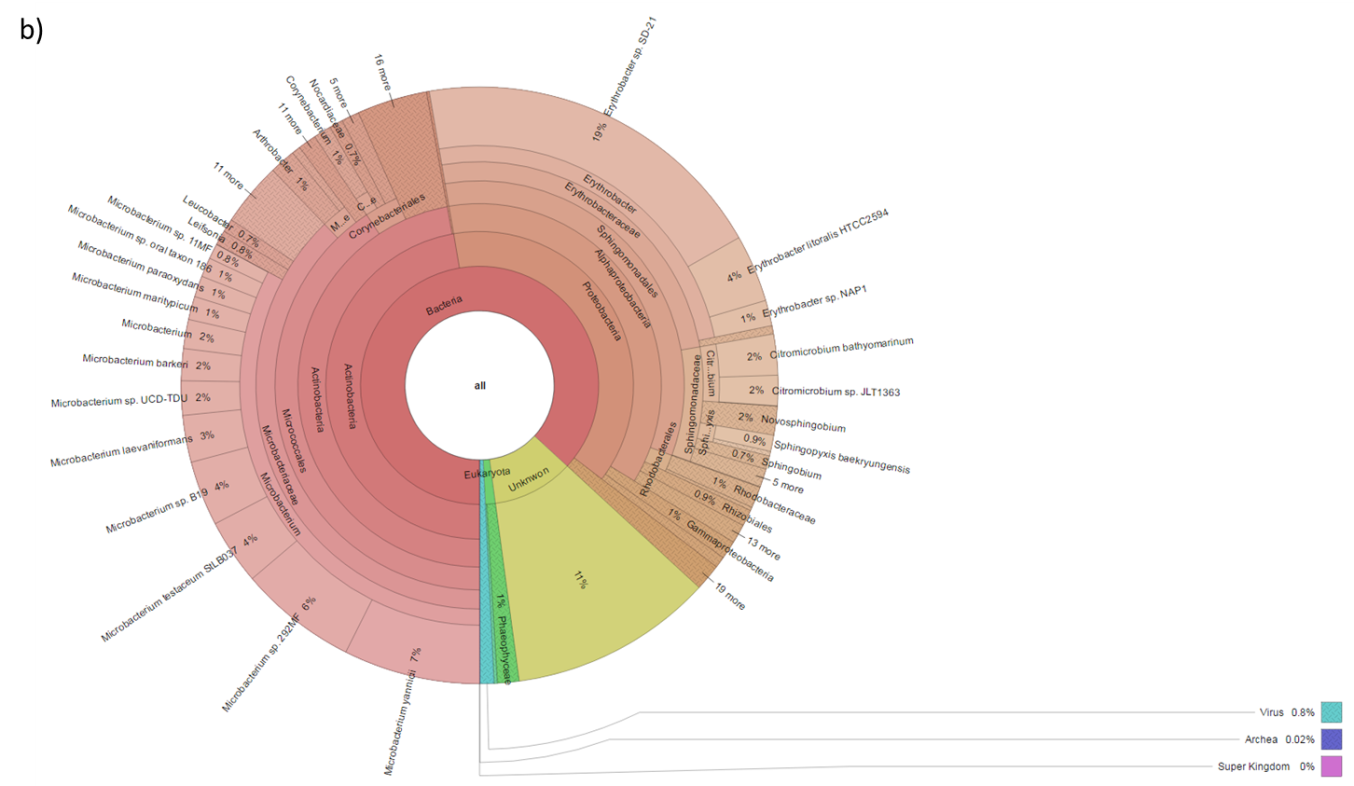

Supplement: Supplementary file 1 [file viruses-09-00047-s001.zip › Supplementary revised/Supplementary_Figure3.docx]
